# Dietary Herbal Leaves Mixture Extract Enhances Growth, Antioxidant Status, and Resistance to *Gyrodactylus malalai* in Heteroclarias Catfish, *Clarias gariepinus ♀* × *Heterobranchus longifilis ♂*

**DOI:** 10.1155/anu/1989752

**Published:** 2025-07-29

**Authors:** Bilal Ahamad Paray, Eijaz Ahmed Bhat, Olaolu O. Fawole, Samuel B. Umma, Ibrahim Adeshina

**Affiliations:** ^1^Department of Zoology, College of Science, King Saud University, P.O. Box 2455, Riyadh 11451, Saudi Arabia; ^2^Microbiology/Molecular Physiology of Prokaryotes, Institute of Biology II, University of Freiburg, Schänzlestraße 1 79104, Freiburg, Germany; ^3^Department of Fisheries and Aquaculture, Ladoke Akintola University, Ogbomoso, Nigeria; ^4^Department of Fisheries and Aquaculture, Federal University Wukari, Wukari, Nigeria; ^5^School of Aquaculture, Oueme Department, National University of Agriculture, Porto Novo, Benin; ^6^Department of Aquaculture and Fisheries, University of Ilorin, Ilorin, Nigeria

**Keywords:** growth, *Gyrodactylus malalai*, herbal mixture, heteroclarias, immunity

## Abstract

*Gyrodactylus malalai* commonly attacks fish gills, which can swiftly infect entire fish stocks and cause both biological and monetary losses. The most popular treatment for *G. malalai* infestations in fish farms is chemotherapy; however, these drugs can have major side effects and are expensive. Novel and ecologically friendly treatments are necessary to treat and control such parasite infestations in fish. Our study examined the use of an herbal leaf mixture extract of *Tridax procumbens*, *Mitrascapus scaber*, *Mucuna pruriens*, and *Carica papaya* (EML) as a functional feed supplement to manage this possible parasite infection. We prepared five distinct isonitrogenous diets (400 g/kg crude protein [CP]) supplemented with 0, 2, 4, 6, or 8 g EML/kg. Juveniles of *Clarias gariepinus* ♀ × *Heterobranchus longifilis* ♂ (heteroclarias, mean weight = 14.5 g) were fed with designated feed six times a day for 56 days until they seemed satisfied. Followed by a 14-day exposure to *G. malalai* (NCBI: txid905034; 40 individuals/L of water), fish in each treatment were monitored closely for any clinical symptoms and mortality. Fish fed EML-enriched diets showed significantly (*p*  < 0.05) improved growth performance, showing trend in a dose-dependent order with an optimum value of 5.94 g EML/kg. Intestinal histomorphometry, digesta pH, viscosity, and short-chain fatty acid (SCFA) were significantly higher (*p*  < 0.05) in EML-fed fish in a dose-dependent manner. Furthermore, an enhanced hematological profile was noticed in fish fed on enriched diet, while urea (UREA), creatinine (CREAT), glucose (GLU), and cholesterol (CHOL) levels decreased in fish fed diets supplemented with EML levels than the control (*p*  < 0.05). Antioxidant status and immune response were promoted in fish fed fortified diet (*p*  < 0.05). Postinfestation survival was highest at 8 g EML/kg (80.00%) compared to control (47.50%). The study concluded that EML improved the hemato-biochemical profile and growth performance of heteroclarias without having any major negative effects, as well as protection against *Gyrodactylus malalai* infestation with an optimum level of 5.94 g/kg. This study is the first to report the use of a standardized herbal leaf mixture extract (EML) comprising *T. procumbens*, *M. scaber*, *M. pruriens*, and *C. papaya* as a practical and functional dietary supplement for the management of *Gyrodactylus malalai* infestation in heteroclarias, documenting novel evidence of the dual role of EML in promoting growth performance and enhancing improving postinfestation survival.

## 1. Introduction

Catfish production has become a huge business across the world, becoming the second most farmed finfish, yielding up to 31,788,000 metric tonnes annually [1]. Catfish, especially heteroclarias, *Clarias gariepinus ♀* × *Heterobranchus longifilis ♂*, are important economic fish and have become a species of special interest due to their fast growth, high-quality flesh, and market value [2], among others. Therefore, several improved production techniques have been developed for its production to reduce culturing periods, feed consumption, and improve growth performance and flesh quality [3, 4], resulting in an intensive farming practice. However, in spite of the success recorded in catfish production, parasite infestations have been hindering its potentialities, especially the emergence of new parasite strains/species and the reoccurrence of old ones [5].

*Gyrodactylus malalai* is a monogenean parasite that commonly affects freshwater fish species [6]. It is a viviparous parasite that attacks the fish by releasing live larvae on the skin and/or gills and rapidly multiplies in the host within 24 h [7]. Its common symptoms are skin discoloration, descaling, and dermatitis. Others include excessive sloughing and skin peeling. *G. malalai* infestation also resulted in skin lesions and injuries, and thus serves as a causative agent to other foreign pathogens and huge mortalities [6, 7]. The presence of *G. malalai* on the gills of African catfish, *Clarias gariepinus*, resulted in histopathological disorder and 19% mortalities [8]. Mortality rates up to 63% were reported in Nile tilapia, *Oreochromis niloticus*, due to *G. malalai* infestation [6, 7, 9 –11]. Therefore, the need to prevent *G. malalai* infestation in fish cannot be overemphasized, and if infestation occurs, quick intervention to manage, control, and treat parasite infestations is required for profitable farming.

Antibiotics and other forms of synthetic drugs are commonly used among farmers to promote growth and control diseases in fish, but it has been discouraged due to the emergence of drug-resistant organisms, residual effects, and environmental degradation, among others [5, 12, 13]. These contentious issues have necessitated the search for environmentally friendly alternatives to promote organic, profitable, and responsible fish farming. In the quest for an alternative to chemotherapeutics, plant materials have been used to promote growth, health, and protection against foreign pathogens [14–16]. Plant materials have been used in tradomedicine from time immemorial, and thus, it has been domesticated in fish farms because of the high presence of active compounds and secondary metabolites, making it an antiparasitic agent. For instance, Adeshina et al. [10] showed that the use of *Mitracarpus scaber* extract stimulated growth, antioxidants, innate immunity, and protection against *G. malalai* infestation in Nile tilapia. *M. scaber* contains high alkaloids, flavonoids, and tannin contents, as well as antimicrobial and antimycotic properties such as gallic acid, 3,4,5-trimethoxybenzoic acid, 4-methoxyacetophenone, 3,4,5-trimethoxyacetophenone, n-octane, 2-hexanol, p-cymene, α and β pinene, which have been shown to stimulate growth, enhance antioxidant enzyme activity, and boost innate immunity [17, 18]. Similarly, *Colossoma macropomum* exposed to 60 mg/L *Piper aduncum* leaf extract was protected against monogenean infestation [19]. Furthermore, studies have shown the stimulatory roles of some botanical herbs against parasites, that is *Euphorbia fischeriana* extract against *D. vastator* in goldfish [20], acetone extract from *Bixa orellana* against *Anacanthorus spathulatus* in tambaqui [21]. Extracts of *Mucuna pruriens*, *Carica papaya*, *Capsicum frutescens*, or *Galla chinensis* against the *Ichthyophthirius multifiliis* [22–24]. *M. pruriens* contains significant levels of L-DOPA, phenolic compounds, and alkaloids, which have been shown to modulate immune responses, stimulate hematopoiesis, and exert antiparasitic [25, 26], while *C. papaya* leaves are rich in papain, alkaloids, flavonoids, and glycosides, known for their anthelmintic and antiparasitic properties [27, 28]. *Tridax procumbens* extract protected Nile tilapia against *Dactylogyrus vastator* infestation [29], which is largely attributed to its high flavonoids, carotenoids, and essential oils, resulting in antimicrobial, wound-healing, and antiparasitic activities [30, 31].

Reports have shown that polyherbal mixtures could perform better than their individuals because bioactive components in each of the plants in the herbal mixture may complement one another using their synergistic potential. So, it looks like extracts from *T. procumbens*, *M. scaber*, *Mucuna pruriens*, and *Carica papaya* leaves could help fish grow faster, boost their immune systems, and keep parasites away [32]. The selection of these plants was based on their well-documented medicinal properties, presence of bioactive compounds in high quantity, and efficacy against various parasites, including *Gyrodactylus* species. While each of these plants individually exhibits antiparasitic and growth-promoting effects, combining them could offer a broad-spectrum synergistic effect that leverages their complementary bioactive profiles, enhancing their overall therapeutic potential. The combined action of their phytochemicals likely produces additive or synergistic effects, enhancing antioxidant defenses, stimulating immune responses, improving nutrient assimilation, and offering broader-spectrum, eco-friendly protection against parasite infestations. Despite these advantages, yet, its application to treat *G. malalai* infestation has not been fully elucidated. Thus, an extract of a mixture of *T. procumbens*, *M. scaber*, *M. pruriens*, and *C. papaya* leaves (EML) was added to diets to examine its influence on growth performance, intestinal histomorphometry, antioxidant status, short-chain fatty acids (SCFAs), immunity of heteroclarias, and protection against *G. malalai* infestation.

## 2. Materials and Methods

### 2.1. Study Period

Following the ethical approval on January 12, 2024, the acclimatization and feeding trial were conducted between March 11 and May 20, 2024. The challenge test was carried out from May 21 to June 3, 2024, while other laboratory and statistical analyses were performed between June 4 and August 30, 2024.

### 2.2. Plant Collection, Identification, and Extraction

The leaves of *T. procumbens* [29], *M. scaber* [10], *M. pruriens*, and *C. papaya* [22] were collected in Ilorin metropolis and authenticated at the Herbarium of the Forest Research Institute of Nigeria (FRIN), Ibadan, Nigeria, before the extract was prepared. Briefly, 5 g of each leaf was air-dried at 35°C before being ground together at a ratio of 1:1:1 into a fine powder using a hand blender. The mixture powder was then cold-extracted in 200 mL of 80% ethyl acetate for 48 h. The filtrate was separated using a No. 1 Whatman paper before the solvent was removed using a rotary evaporator (Model: SKU:418U1309, Genser Scientific Powervap, Barneved, NL) [20] to produce an extract of leaves of *T. procumbens*, *M. scaber*, *M. pruriens*, and *C. papaya* (EML). The yield was 1.75 ± 0.04 g EML. The EML yield was repeated until the required quantity was achieved and stripped into a glass jar and kept at −20°C until required.

### 2.3. Phyto-Chemical and Phyto-Constituents' Analyses

The EML was examined for tannins [33], terpenoids [34], steroids [35], flavonoids [36, 37], alkaloids [37], and saponins [36, 37], while the bioactive compounds were evaluated using gas chromatography (GC; Agilent 5975, Avondale, PA, USA)/mass spectrometry (MS; Agilent 7890A, Avondale, PA, USA) containing a 5-meter-long HP column (0.25 cm internal diameter). Briefly, Agilent 190915-433HP-5M, 5% phenylmethylsilox (30 m × 250 × 0.25 μm) operated at an ionization energy of 70 eV, with splitless injector (at 300°C) and 1.0 µm film thickness [37]. An auto sampler was used to inject 1 µL of each sample. The oven temperature was programed from 35 to 300°C, held at 35°C for 5 min, then at a rate of 20°C per min to 250°C for 5 min using helium carrier gas at a flow of 1 mL per min. The samples were run using full scan with a range of 50–750 mass units, and recorded using HP ChemStation system [38]. The extract components were identified by comparing their relative retention times and mass spectra with those of authentic samples (analytical standards from the database) as described by Bayoub et al. [39]. The library database (NIT II), selecting only those structures that reached 90% or more probability, made the structural assignments. The metabolites and phytoconstituents were shown in Tables 1 and 2, respectively.

### 2.4. Experimental Diet and Culture Technique

A control diet (400 g/kg crude protein [CP]) was fortified with EML at 0 (control), 2, 4, 6, or 8 g of EML/kg of diet to form five experimental diets (Table 3). The dosage levels were based on the dose responses in previous works [10, 22, 24, 29]. The diets were mixed with 100 mL of sterile water per kg to form dough. The dough was then pelleted through a 2 mm diameter size and dried at ambient temperature for 24 h. Then, the diets were kept at −20°C until required. Also, the diets were reformulated every fortnight to prevent nutrient loss and EML degeneration.

Heteroclarias juveniles (12.2 ± 0.11 g) were obtained from a registered farm in Ilorin, Nigeria, and acclimatized for 2 weeks in 1 m^3^ aquaria. During this period of 2 weeks, the fish were fed with the basal diet. Then, 300 fish (mean = 14.5 g) were distributed into 20 aquaria (15 fish per aquarium) with 100 L capacity (i.e, 5 treatments with 4 replicates) connected to de-chlorinated water in a completely randomized design manner using a random table. The fish were fed one of the experimental diets for 56 days to apparent satiation six times a day (8:00, 10:00, 12:00, 14:00,16:00, and 18:00 h).

The water was monitored every day for dissolved oxygen (DO) and temperature using a dual-parameter EcoSense meter (YSI, Model No. DO200A, China) and pH using a digital pH meter (Model Photoic 20, Labtech International Ltd). The values of the water temperature, DO, and pH were between 25.3 and 28.3°C, 5.8 and 6.6 mg/L, and 7.3 and 7.8, respectively. These values were within the recommended values required for culturing catfish [40]. Then, the fish from each aquarium group were counted and weighed using a sensitive scale (Global Ltd, Model No. GS-B1, China). Fish were anesthetized for 5 min in 30 mg/L of sodium bicarbonate buffered with tricaine methane sulfonate (MS222, 30 mg/L, Syndel, Ferdale, Washington, USA) before weighing on the scale. Growth parameters were measured as follows:

  $$\begin{array}{c} \text{Body weight gain}\left(\text{BWG},\text{ g}\right)=\text{ final body weight}\left(\text{FBW},\text{ g}\right)-\text{initial body weight}\left(\text{IBW},\text{ g}\right), \end{array}$$

  $$\begin{array}{c} \text{Specific growth rate}\left(\text{SGR};\%{\text{ day}}^{-1}\right)=100\left[\text{Ln FBW}\left(\text{g}\right)-\text{ Ln IBW}\left(\text{g}\right)\right]/\text{duration of trial}, \end{array}$$

  $$\begin{array}{c} \text{Feed intake}\left(\text{FI g}/\text{fish}\right)=\text{ total feed distributed}/\text{number of fish}, \end{array}$$

  $$\begin{array}{c} \text{Feed conversion ratio}\left(\text{FCR}\right)=\text{ FI}\left(\text{g}\right)/\text{ BWG}\left(\text{g}\right), \end{array}$$

  $$\begin{array}{c} \text{Fish survival rates}\left(\text{SRs}\right)\left(\%\right)=100\left(\text{final number of fish}/\text{initial number of fish}\right). \end{array}$$

### 2.5. Intestinal Histomorphometry

The three fish selected were then euthanized using the two-step procedure of the American Veterinary Medical Association [41], that is, the fish were immersed in 1000 mg/L MS222 and the secondary penetrating captive bolt method. The fish were aseptically dissected, and the intestines were prepared for histomorphometry examination according to Bancroft and Gamble [42]. In brief, the samples were preserved in 20% buffered formalin (Sigma–Aldrich, St. Louis, MO) and then Bouin's fluid [43]. The tissues were then dehydrated in 70%, 80%, 90%, 95%, and 100% ethanol before being embedded in paraffin wax and cut into sections. Then, 5 μm sections of the specimens were dewaxed in xylene before being hydrated in 100%, 95%, 90%, 80%, and 70% ethanol. Then, the specimens were stained with hematoxylin and eosin (Sigma–Aldrich, St. Louis, MO) and observed under a light microscope (Olympus CX21, Japan). The villi width (VW; μm), villi length (VL; μm), and cryptal depth (CP; μm) were measured with the aid of calibre meter. Area of absorption (AA; μm^2^) was estimated as area of absorption (μm^2^) = VL × VW (μm).

### 2.6. Evaluation of Digesta pH, Viscosity, and SCFAs

From three fish, the digesta pH was measured in situ by inserting the probe of the pH meter (Photoic 20, Labtech International Ltd., Heathfield, UK) into the intestinal digesta of each fish, and the readings were recorded. Viscosity was measured by centrifuging the feces at 28°C and 3000 x *g* for 10 min (model: LC400, Joanlab Laboratories, China). Then, the supernatant was placed in a viscometer (NDJ-9S, RaeSung Inc., India) using a 50.0 per sec shear rate to assess viscosity (Centipoise, cP) [44].

The SCFAs (acetic, propionic, and butyric acids) were examined using GC (Agilent 7890A) (Avondale, PA, USA) equipped with an HP column of 5 m long (Agilent 190915-433HP-5M) operated at ionization energy of 70 eVa flame ionization detector, after acidification with 1 Mo-phosphoric acid p.a. (Ref. 100,573, Merck) and fortification with a mixture of free volatile acids (Ref. 46,975, Supelco). In order to separate the analytes in a chromatographic run of 11.5 min, an aliquot of 1 μL of each sample was injected (split ratio of 40:1) using helium as a carrier gas with a linear velocity of 42 cm/s. The initial column temperature was 40°C, and the injector and detector temperatures were 250°C and 300°C, respectively. Starting at 40°C min 1, the column temperature ramp progressed from 40 to 120°C, then from 120 to 180°C at 10°C min 1, and finally from 180 to 240°C at 120°C min 1, maintaining the temperature at 240°C for an additional 3 min. The aforementioned conditions were used to analyze calibrated dilutions of the WSFA-2 standard (Ref. 47,056, Supelco) and glacial acetic acid (Ref. 33,209, Sigma–Aldrich) in order to quantify the analytes. The software GC solution v. 2.42.00 (Shimadzu, Kyoto, Japan) was used to identify and integrate the peaks.

### 2.7. Assessment of Hematological and Biochemical Markers

Three fish from each aquarium were anesthetized for 5 min in 30 mg/L of sodium bicarbonate buffered with tricaine methane sulfonate (MS222, 30 mg/L, Syndel, Ferdale, Washington, USA) [45]. Then, with the aid of a 2 mL syringe and needle, blood samples were drawn from the caudal veins of fish and divided into two. The first part was dispensed into bottles that contained 20 U/L lithium heparin anticoagulant at room temperature to measure hematological indices, that is, packed cell volume (PCV; %), red blood cells (RBCs; × 10^6^/µL), white blood cells (WBCs; × 10^3^/µL), and platelets (PLTs; × 10^6^/µL) according to Van Kampen and Zijlstra [46], while hemoglobin (Hb; g/dL) was measured using the Brown [47] method. In addition, using the Wright–Giemsa stain method, differential counts, that is, lymphocytes (LYMs; %), heterocytes (HETs; %), monocytes (MONs; %), eosinophils (EOSs; %), and basophils (BASs; %), were estimated. However, the second portion was left to clot before centrifuging (model: LC400, Joanlab Laboratories, China) it at 35°C and 5000x *g* for 10 min to retrieve the serum with the aid of a micropipette. Then, aspartate aminotransferase (AST; IU/mL) (catalogueueue number = AS8005), alkaline phosphatase (ALP; IU/mL) (catalogueueue number = AP307), and alanine aminotransferase (ALT; IU/mL) (catalogueueue number = AL3801) were measured using the colorimetric method using Randox commercial kits (Randox Laboratories Ltd. Crumlin, County Antrim, United Kingdom) [48, 49].

### 2.8. Determination of Serum Biochemistry Profiles of Heteroclarias Fed With EML-Based Diets

From the three fish, urea (UREA) was determined using Randox UR 1068 kits. 10 µL of the sample was taken using micro-pipette, mixed with 100 µL of reagent 1, and incubated for 15 min at 37°C. The absorbance of the sample and standard was read on photospectrometer (SM23A). The color of the reaction was stable for at least 8 h. The values were calculated using UREA concentration (mg/dL) = $\frac{A_{\text{sample}}}{A_{\text{standard}}}\times \text{standard concentration}$ [50]. Standard concentration = 79.81 mg/dL. $\text{Urea}+{\text{H}}_{2}\text{O}\vec{\text{Urease}}2{\text{NH}}_{3}+{\text{CO}}_{2};{\text{NH}}_{3}+\text{hypochlorite}+\text{phenol}\to \text{indophenol}\left(\text{bluecompound}\right)$. Creatinine (CREAT) was determined using Randox CR 510 kits. Exactly 0.1 mL of the sample was taken using micro-pipette, mixed with 1.0 mL of reagent 1, and after 30 s, the absorbance *A*_1_ values of the standard and sample were read on photospectrometer (SM23A). Exactly 2 min later, absorbance *A*_2_ values of standard and sample were read. The values were calculated using *A*_2_ − *A*_1_ = ΔAsample, *A*_2_ − *A*_1_ = ΔStandard. CREAT (mg/dL) = $\frac{\Delta A_{\text{sample}}}{\Delta A_{\text{standard}}}\times \text{standard concentration}\times 50$. Standard concentration = 2.06 mg/dL. The amount of complex formed is directly proportional to the CREAT concentration [50, 51]. Glucose (GLU) was determined using Randox GL 364 kits. Exactly 10 µL of the sample was taken using micro-pipette, mixed with 1000 µL of reagent 1, and incubated for 25 min at 25°C. The absorbance values of the sample and standard were read against reagent blank within 60 min on photospectrometer (SM23A). The values were calculated using GLU concentration (mg/dL) = $\frac{A_{\text{sample}}}{A_{\text{standard}}}\times$standardconcentration. Standard concentration = 5.00 mg/dL. $\text{Glucose}+{\text{O}}_{2}+{\text{H}}_{2}\text{O}\vec{\text{GOD}}\text{gluconicacid}+{\text{H}}_{2}{\text{O}}_{2};2{\text{H}}_{2}{\text{O}}_{2}+4-\text{aminophenazone}+\text{phenol}\vec{\text{POD}}\text{quinoneimine}+4{\text{H}}_{2}\text{O}$. The values were standardized using Randox calibration serum level 3 traceable to GLU reference materials NIST 917b and NIST 965a. Cholesterol (CHOL) was determined using Randox CH 200 kits. 10 µL of the sample was taken using micro-pipette, mixed with 1000 µL of kit reagent and incubated for 5 min at 37°C. The absorbance values of the sample and standard were read against the reagent blank within 60 min on photospectrometer (SM23A). The values were calculated using CHOL concentration (mg/dL) = $\frac{\Delta A_{\text{sample}}}{\Delta A_{\text{standard}}}\times \text{standard concentration}$ [51]. Standard concentration = 208 mg/dL. $\text{Cholesterolesther}+{\text{H}}_{2}\text{O}\vec{\text{Cholesterol esterase}}\text{ Cholesterol}+\text{Fattyacids}$. $\text{Cholesterol}+{\text{O}}_{2}\vec{\text{Cholesterolexidase}}\text{ Cholesterol}-3-\text{one}+{\text{H}}_{2}{\text{O}}_{2}$

H_2_O_2_ + phenol + 4 − Aminoantipyrine  peroxidase quinoneimine + 4H_2_O. The CHOL was determined after enzymatic hydrolysis and oxidation. The indicator quinoneimine was formed from hydrogen peroxide and 4-aminoantipyrine in the presence of phenol and peroxidase [14].

### 2.9. Assessment of Antioxidant Response of Heteroclarias Fed With EML-Based Diets

From serum, antioxidant activities were measured with the aid of diagnostic kits (Invitrogen ThermoFisher Scientific Inc.). Superoxide dismutase (SOD; IU/mL; catalog number = EIASODC), catalase (CAT; IU/mL; catalog number = EIACATC), and malondialdehyde (MDA; nmol/L; catalog number = EEA015) were determined according to the methods of McCord and Fridovich [52] and Aebi [53], respectively.

### 2.10. Determination of Immune Response of Heteroclarias Fed With EML-Based Diets and Susceptibility to Parasitic Infestation

Respiratory burst activity (RBA; µmol) was measured using nitroblue tetrazolium dye [54], while lysozyme (LYZ; U/mg protein) enzymes were measured using the turbidimetric method [55]. *G. malalai* (NCBI:txid905034) acquired from the Department of Microbiology, University of Ibadan, Ibadan, was used for the challenge test [10]. In brief, from each aquarium, 10 fish were denied feed for 24 h before being exposed to a *G. malalai* (40 individuals/L) solution [10]. The feeding was returned for 14 days, as used in the growth study.

### 2.11. Statistical Analysis

The data (see supporting information file) obtained was first tested for homogeneity of variances and normality of distribution using Kolmogorov–Smirnov and Bartlett tests. After that, the data was analyzed using one-way analysis of variance. Means that were significantly different were separated using Tukey's test. Furthermore, the optimum inclusion level of EML in the diet of heteroclarias was determined using polynomial regression with the social package for social science (SPSS) version 20, Richmond, VA, USA [56]. The best-fit model was selected based on R-squared (*R*^2^), that is, how much variance in the dependent variable is explained by the model.

## 3. Results

### 3.1. Growth Performance and Nutrient Utilization of Heteroclarias Fed EML-Based Diets

**Figure 1:**
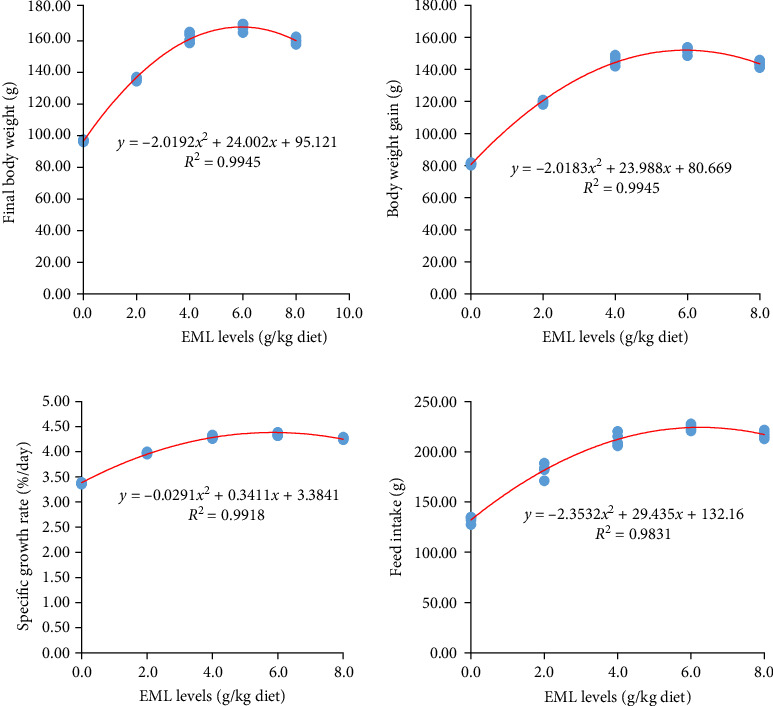
The relationship between final body weight (g), body weight gain (g), specific growth rate (%/day), and feed intake (g/fish) of heteroclarias and different levels of dietary EML. ^1^Number of replicates is 4. ^2^EML = Extract of *T. procumbens*, *M. scaber*, *M. pruriens*, and *C. papaya* mixture leaves.

Table 4 depicts the growth performance and nutrient utilization of heteroclarias fed dietary EML for 56 days. Significant (*p*  < 0.05) increased growth performance was observed in fish fed fortified diets. FBW, BWG, SGR, FI, and SGR were higher in fish fed a diet enriched with 6.0 g EML/diet than in the fish fed on a control diet (*p*  < 0.05) (Table 4). Conversely, FCR was greatly (*p*  < 0.05) reduced in fish fed a supplemented diet compared to the control group. Fish fed with the control diet had the highest FCR, while the least FCR was noticed in fish fed on 6.0 g EML/diet. The quadratic regressions indicated the relationship between FBW, BWG, SGR, and FI of heteroclarias and different levels of dietary EML are best as *y* = −2.0192*x*^2^ + 24.002*x* + 95.121(*R*^2^ = 0.9945), *y* = −2.0183*x*^2^ + 23.988*x* + 80.669(*R*^2^ = 0.9945), *y* = −0.0291*x*^2^ + 0.3411*x* + 3.3841(*R*^2^ = 0.9918), and *y* = −2.3532*x*^2^ + 29.435*x* + 132.16(*R*^2^ = 0.9831), respectively (Figure 1). The curves revealed that the most appreciated EML inclusion level for optimum fish growth performance is found to be 5.94 g EML/kg diet revealing the dosage that will produce appreciable and optimum growth with minimum EML inclusion. However, dietary EML did not significantly (*p* = 0.405) affect SR, which revealed that EMLLE has no harmful effects on the well-being of fish (Table 4).

### 3.2. Intestinal Histomorphometry of Heteroclarias Fed EML-Based Diets

**Figure 2:**
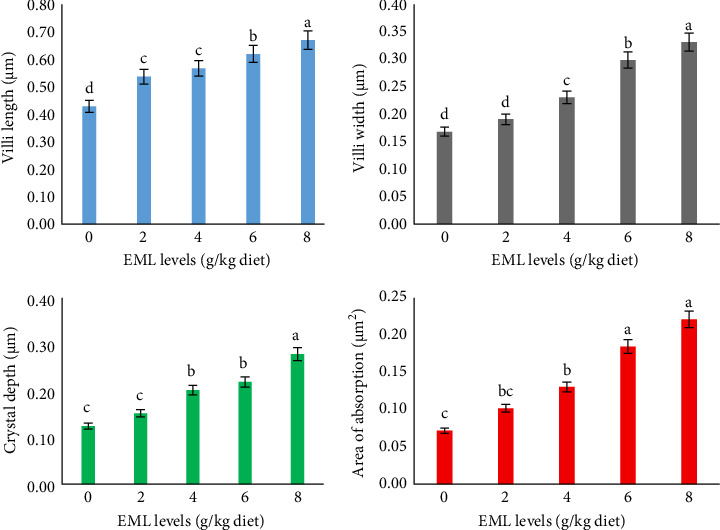
Intestinal histomorphometry of heteroclarias fed with diets containingvarious levels of EML for 56 days^1^. Values are presented as mean ± standard deviation. Means in the same row with difference superscript are statistically significantly different (*p*  < 0.05). ^1^Number of replicates is 4. ^2^EML = Extract of *T. procumbens*, *M. scaber*, *M. pruriens*, and *C. papaya* mixture leaves.

The intestinal histomorphometry of heteroclarias fed on EML-based diets was presented in Figure 2. There were significant (*p*  < 0.05) higher VL, VW, and CD in fish fed enriched diets than in the control group. In a dose-dependent manner, AA was greatly higher in fish fed fortified diets; the values ranged between 0.07 and 0.22 in fish fed a control diet and an 8 g EML/kg diet, respectively (*p* = 0.001; Figure 2).

### 3.3. Evaluation of Digesta pH, Viscosity, and SCFAs

Table 5 depicts the digesta pH, viscosity, and SCFA of heteroclarias fed with an EML-based diet. The digesta pH, acetic, propionic, and butyric (*p*  < 0.05) acids were significantly elevated in fish fed fortified diets compared to the fish fed with the control diet. However, dietary EML did not significantly (*p* = 0.790) affect viscosity, and propionic acid was not significantly affected by diet (Table 5).

### 3.4. Hemato-Biochemical Profile of Heteroclarias Fed EML-Based Diets

**Figure 3:**
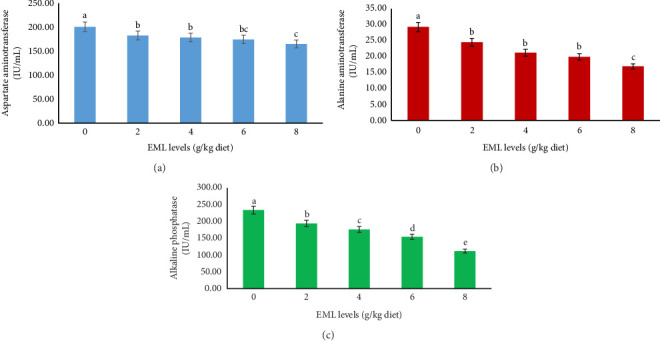
Hepatic parameters (a) aspartate aminotrasferase (IU/mL), (b) alanine aminotrasferase (IU/mL), and (c) alkaline phosphatase (IU/mL) of heteroclarias fed with diets containing various levels of EML for 56 days^1^. Bars are presented as mean ± standard deviation. Bars with difference superscript are statistically significantly different (*p*  < 0.05). ^1^Number of replicates is 4. ^2^EML = Extract of *T. procumbens*, *M. scaber*, *M. pruriens*, and *C. papaya* mixture leaves.

Hematological profiles of heteroclarias fed on dietary EML were significantly different (*p*  < 0.05; Table 6). A steady increase in the level of PCV, Hb, RBC, WBC, and PLT in fish fed enriched diets when compared to the fish fed with the control diet in a direct proportional to the increase in the level of EML (Table 6). Further, in a dose-related manner, an increase in LYM and MON and a decrease in HET were recorded, but dietary EML did not significantly alter the EOS and BAS levels in heteroclarias. In addition, significant reductions (*p*  < 0.05) in ALP, AST, and ALT activities were recorded in fish treated with dietary-EML (Figure 3). The minimum values of ALP, AST, and ALT were noticed in fish nourished with an 8 g EML/kg diet level, while the fish fed on the control diet had the least values (Figure 3).

### 3.5. Serum Biochemistry Profiles of Heteroclarias Fed With EML-Based Diets

Serum biochemistry profiles were markedly (*p*  < 0.05) varied in heteroclarias fed with dietary EML, as shown in Table 7. The variations observed in the fish were directly proportional to the EML inclusion levels. Fish fed on the control diet had the highest UREA, CREAT, GLU, and CHOL, while the lowest values were recorded in fish fed with 8 g EML/kg diet.

### 3.6. Antioxidant Status of Heteroclarias Fed With EML-Based Diets

Table 8 demonstrates that the inclusion of EML in the diets of heteroclarias resulted in a significant (*p*  < 0.001) increase in the levels of SOD and CAT but a decrease in MDA levels. These differences were found to be in the same trend as the EML supplementation levels. Specifically, fish fed a diet containing 8 g EML/kg exhibited the highest levels of SOD and CAT but the lowest MDA level, while fish fed on the control diet possessed the least SOD and CAT with the highest MDA.

### 3.7. Immune Response Profiles and Postinfestation Survival of Heteroclarias Fed With EML-Based Diets

**Figure 4:**
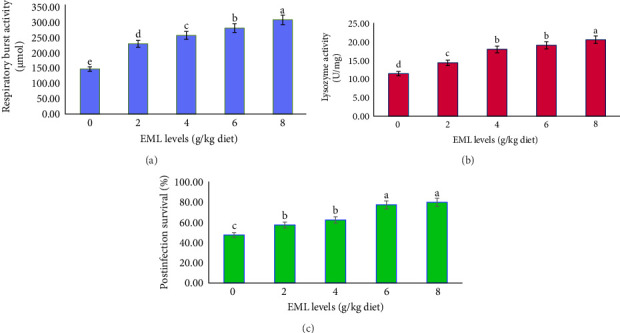
Immune response and postinfection survival of heteroclarias fed diets containing various levels of EML. (a) Respiratory burst activity (µmol), (b) lysozyme activity (U/mg), (c) postinfection survival (%). Bars are presented as mean ± standard deviation. Bars with difference superscript are statistically significantly different (*p*  < 0.05). ^1^Number of replicates is 4. ^2^EML = Extract of *T. procumbens*, *M. scaber*, *M. pruriens*, and *C. papaya* mixture leaves.

Figure 4 depicts a notable elevation in the immune response of heteroclarias fed an EML-enriched diet (*p*  < 0.05). Significantly higher RBA and CAT were observed in fish fed with 8 g EML/kg diet, while fish fed a control diet had the least. Similarly, postinfestation survival was greatly (*p*  < 0.05) induced by dietary EML, as the highest postinfestation survival (80.00%) was higher in fish fed on 8 g EML/kg than in the control group (47.50%) (*p*  < 0.05; Figure 4).

## 4. Discussion

The demand for fish is increasing on a daily basis, and the production of fast-growing fish like heteroclarias could help in promoting food security. Therefore, measuring growth parameters is a widely accepted tool to ensure a profitable fish farming business without compromising fish quality. The findings of this study revealed a boosted growth performance in heteroclarias fed on graded EML-based diets. This increased growth underlines the potential and usability of EML as a natural growth promoter. Higher FBW, BWG, and SGR caused by dietary EML signify that its supplementation in the fish diet influences digestion, assimilation, utilization, and metabolic activities. Previously, African catfish [57], Japanese seabass, *Lateolabrax japonicus* [58], and European eel, *Anguilla anguilla* [59], demonstrated improved growth when fed diets enriched with different herbal mixtures. Also, in common carp, *Cyprinus carpio* [60], and olive flounder, *Paralichthys olivaceus* [61], fed a diet containing a 5 g/kg mixture of oak acorn, coriander, and common mallow extract and *Cnidium officinale*, *Massa medicata fermentata*, *Crataegi fructus*, and *Artemisia capillaries*, respectively, enhanced a higher growth performance. In addition, higher FI and lower FCR observed in fish fed fortified diets indicate that the feed is more palatable to the fish, and feed efficiency was higher than what was observed in fish fed the control diet. These observations are linked to the bioactive compounds' presence in EML, such as α-Pinene, β-Sitosterol, p-Cymene, caryophyllene, and phytol, which stimulate appetite by enhancing feed taste, aroma, and palatability, and improve digestion and metabolism, leading to better growth [62]. The study establishes that these plant materials greatly work together and complement each other, as the active compounds show no antagonistic activity on the growth of heteroclarias as well as no deleterious effects supported by the SR. The study agreed with the findings of Ji et al. [61], who reported no significant difference in the SR of olive flounder fed with dietary EML. The positive correlations shown between growth performance (FBW, BWG, SGR, and FI) and EML levels indicated the influence of EML on heteroclarias is strong and reliable.

In this study, dietary EML greatly evoked VL, VW, CD, and consequently AA in the intestines of heteroclarias, especially at an 8 g/kg diet. These results revealed that the mode of action of EML in the intestines of heteroclarias is due to its ability to induce longer and thicker villi and increased AA in the intestines, which resulted in better nutrient absorption. Studies have shown that improvement of intestinal morphology creates a chance of increased nutrient uptake and subsequent higher growth performance and feed utilization [63–66], which is in line with the observation of the current study. In a similar study, African catfish [14, 16], Nile tilapia [66], and Gilthead seabream (*Sparus aurata*) [63] fed on dietary *Eugenia caryophyllata* buds and *Ocimum gratissimum* leaves extracts and astragalus polysaccharide, respectively, had elevated VL, VW, CD, and AA than their counterparts in the control group.

In addition, dietary EML significantly increased digesta pH, acetic, propionic, and butyric acid levels. When the gut bacteria fermented the carbohydrates, SCFAs will be produced [67, 68], which will influence microbial activity indirectly, leading to improved growth performance and feed efficiency [69 −73 ]. This study revealed that EML positively influences the production of SCFAs, with the increase in the level of acetic, propionic, and butyric acid levels in EML-dose-dependent order. Studies revealed that SCFAs improve intestinal cell walls to permit more nutrient absorption through altering the junction proteins between intestinal cells, leading to an improvement in gut health [71–73].

Knowledge of blood profile is an important tool in understanding the effects of supplemented feed on pathophysiological condition and nutritional status of fish [57]. PVC, Hb, RBCs, WBCs, and PLT were all significantly increased in a dose-dependent manner in heteroclarias fed dietary EML. The study also found that fish fed EML-based diets had higher blood oxygen transportation, which is especially helpful for fish in environments where oxygen levels are low and/or during activities that require more oxygen, thus helping them cope with temperature fluctuations. Additionally, high PCV can be linked to robust fish health, better growth performance, and enhanced immunity. Similarly, as RBCs carry oxygen, a higher number of them enhances the fish's capacity to meet oxygen requirements and bounce back fast from infections and other stressors. On the other hand, WBCs are essential in the fight against pathogenic threats. Therefore, a rise in WBCs suggests a strong immune system that can combat infections. The current investigation showed no discernible changes in the WBC level, indicating that the fish were not exposed to any infections. PLT contributes to the development of clots. An increase guarantees quick wound healing and effective blood coagulation. Improved hematopoiesis and increased oxygen-carrying capacity are suggested by the rise in PCV, Hb, RBCs, and PLT counts. These findings may support improved metabolic activity and general physiological functioning. When given dietary-herbal mixes, common carp [60] and African catfish [57] showed comparable outcomes. In fish fed EML, the increase in LYM and MON and the corresponding drop in HET further suggests a stronger immune system and possibly less inflammation brought on by stress.

Protein metabolism enzymes like AST, ALT, and ALP are commonly used to assess liver health issues because they are produced as a result of liver malfunction [74]. ALP catalyzes the hydrolysis of phosphate esters and participates in phosphorus metabolism. AST and ALT facilitate the transmission of an amino group from aspartate to α-ketoglutarate for the formation of glutamate and oxaloacetate. In this study, it was revealed that addition of EML upto 8 g EML/kg could reduce hepatic stress and promote liver health. The reduction in their levels suggests that the liver is not undergoing significant damage or stress and thus reflects normal physiological conditions. Fawole et al. [57] reported that African catfish fed dietary polyherbal mixture had reduced liver enzyme activities and that it had no hepatotoxic effect on fish.

As regards serum biochemistry, our study established a significant reduction in serum biochemistry profiles of heteroclarias fed with graded EML levels. Remarkably, fish fed on the control diet had the highest UREA, CREAT, GLU, and CHOL levels, whereas the corresponding lowest values were observed in fish with an 8 g EML/kg diet. In a dose-dependent manner, improved metabolic activities due to EML supplementation could be linked to the presence of bioactive compounds in it, leading to reduced physiological stress and enhanced health status. This observation was similar to what was noticed in African catfish [57] and Nile tilapia [75] fed a dietary herbal mixture. On the contrary, no significant influence of turmeric extract was noticed on UREA and CREAT of *Pangasius bocourti* as reported by Nguyen et al. [76]. The differences in these results may be linked to the ability of each fish species to respond to phytogenic additives as well as environmental factors. In contrast, our study indicates that EML influences GLU metabolism and modulates nitrogenous waste products, implying a broader physiological impact on heteroclarias.

Because they play a crucial role in preserving the proper redox equilibrium and neutralizing reactive oxygen species (ROS) in biological systems,

SOD, CAT, and MDA play essential roles in maintaining a redox balance and neutralizing ROS in fish. CAT and SOD convert H_2_O_2_ and O_2_^−^ into H_2_O and O_2_, which eventually leads to protection from ROS damage. An elevated SOD and CAT noticed in this study indicated that dietary EML significantly enhanced antioxidant defenses and boosted cellular health in heteroclarias, which was mechanistically attributed to the presence of flavonoids, phenolic compounds, alkaloids, and terpenoids in the herbal mixture. These bioactive compounds are known to exhibit strong antioxidant potential through direct and indirect mechanisms of action. The flavonoids and phenolics present in EML directly scavenge ROS by contributing electrons to unstable free radicals, stabilizing them and preventing oxidative cellular damage and its accumulation [77, 78]. However, a reduced MDA level recorded in this study further reiterates EML's antioxidant ability, which is in agreement with [32] and [57], who reported that lower MDA caused by a diet enriched by phytobiotic extracts increases the ability of fish to withstand stress but enhancing enzymatic antioxidant defenses, EML minimizes oxidative degradation of lipids, leading to a significant reduction in MDA, a major biomarker of lipid peroxidation. As shown in *T. procumbens* extracts exhibiting significant radical scavenging activity [77], *C. papaya* leaves have demonstrated potent antioxidant effects by mitigating oxidative damage in biological systems [78]. The phenolic compounds in *M. pruriens* also contribute by enhancing antioxidant enzyme activities in fish [79]. While the alkaloids in *M. scaber* have been reported to upregulate antioxidant enzymes via modulation of intracellular redox signaling [80].

The increased RBA and LYZ activity in this study, especially at the 8 g EML/kg diet level, indicate that the fish's immune defenses against pathogens are strengthened by EML fortification, most likely through the modulation of innate immune pathways. Prior studies have demonstrated a significantly improves their LYZ and RBA status in African catfish fed *Eugenia caryophyllata* buds extract [14], *Ocimum gratissimum* leaves extract [16], *M. scaber* leaves extract [32], and herbal mixture [57]. Higher RBA and LYZ levels improve the capacity to swiftly and efficiently eradicate infections and signify a strong and active innate immune response. An enhanced antiparasitic defense mechanism is reflected in the LYZ, an antimicrobial enzyme that breaks down the β-1,4-glycosidic link in peptidoglycan to destroy the bacterial cell wall.

The protective effects of EML against parasite threats are highlighted by the increase in postinfestation survival in fish fed EML-based diets, with the maximum survival rate seen in the fish fed 8 g EML/kg diet. This result is consistent with the noted improvements in immunological responses, indicating that EML fortification could increase heteroclarias' resistance to parasite infections. The results have encouraging ramifications for aquaculture disease management since they show that EML supplementation not only enhances growth and health metrics but also helps to lower susceptibility to parasitic mortality. Similar investigations showed that grass carp fed 4 g/kg extract from an herbal mixture were protected against infection by *I. multifiliis*, with a survival rate of up to 71.1% [81–83]. Different herb combinations were the cause of the difference in the postinfestation findings. In African catfish exposed to *D. vastator* infestation, 6 g/kg of *T. procumbens* leaf extract increased survival to 90.0% when each plant material of EML was treated separately [29]. Additionally, 200 g/kg of *M. pruriens* extract reduced gold fish mortality by 90% against *I. multifiliis* [22], 200 g/kg of *C. papaya* extract produced protection up to 90% after *I. multifiliis* infestation in gold fish [22], and 6 g/kg of *M. scaber* leaves extract increased postinfestation survival of Nile tilapia against *G. malalai* to 95.0% [10]. Herbal mixtures have shown promise as agents since they provide better protection against parasites at lower dosages than single herbal extracts, which have high dosages. This further implies that the active ingredients in EML are bioavailable and have a good synergistic interaction, which led to improved protection and a lower degree of inclusion.

## 5. Conclusion

This study concluded that herbal blend of leaf extracts from *Tridax procumbens*, *Mitracarpus scaber*, *Mucuna pruriens*, and *Carica papaya* improved the hemato-biochemical profile and growth performance of heteroclarias without major negative effects. Furthermore, juvenile heteroclarias showed immunostimulatory and antioxidative responses to dietary EML. Notably, adding EML to the feed at the optimum dose of 5.94 g/kg improved the heteroclarias' resistance to *Gyrodactylus malalai* infestation, thus offers a sustainable alternative to chemotherapeutics and reducing reliance on synthetic drugs in aquaculture. However, histopathological evaluations should be carried to confirm the long-term safety of EML in heteroclarias.

## Figures and Tables

**Table 1:** Quantitative analysis of secondary metabolites in the EML^a^.

Parameter	Value (mg 100 g^−1^)
Alkaloids	350.3
Flavonoids	72.4
Saponins	49.9
Steroids	114.3
Tannins	63.5
Terpenoids	702.6

^a^EML = Extract of *T. procumbens*, *M. scaber*, *M. pruriens*, and *C. papaya* mixture leaves.

**Table 2:** Selected phyto-constituents identified in EML^a^.

Name of compound	RT (min)	MF	MW (g mol^−1^)	Conc. (%)	QP (%)
Bifemelane	23.17	C_18_H_23_NO	269.4	2.1	98
α-Pinene	9.72	C_10_H_16_	136.2	2.4	99
β-Sitosterol	20.13	C_29_H_50_O	414.7	1.1	96
p-Cymere	26.19	C_10_H_14_	134.2	2.4	89
Cadimene	23.15	C_15_H_26_	206.37	1.4	93
Caryophyllene	22.67	C_15_H_24_	204.35	0.2	98
Eicosanoic acid	22.81	C_20_H_40_O_2_	312.5	1.1	78
Octadecanoic acid	22.84	C_18_H_36_O_2_	284.5	4.3	56
Salicyclic acid	1.87	C_7_H_6_O_3_	138.12	5.7	98
Phytol	25.31	C_20_H_40_O	296.5	7.3	80
Oleic acid	27.71	C_18_H_34_O_2_	282.5	1.1	94
Tricosane	23.76	C_23_H_48_	324.6	2.7	88
Protocatehuic acid	11.12	C_7_H_6_O_4_	154.12	2.3	76
p-Coumaric acid	11.89	C_9_H_8_O_3_	164.16	0.7	99
Caffeic acid	13.03	C_9_H_8_O_4_	180.16	4.1	97
Kaempferol	19.62	C_15_H_10_O_6_	286.24	3.7	79
Quercetin	20.86	C_15_H_10_O_7_	302.23	1.1	92
Chlorogeric acid	20.01	C_16_H_18_O_9_	354.31	4.8	80
Campesterol	35.21	C_28_H_48_O	400.70	3.2	65
Stigmasterol	35.55	C_29_H_48_O	412.70	4.1	93
17-Octadecynoic acid	4.29	C_18_H_32_O_2_	280.40	2.1	99
Neophytadiene	22.43	C_20_H_38_	278.50	2.7	91
n-Hexadecanoic acid	23.57	C_16_H_32_O_2_	256.42	1.3	68
Undecane	11.19	C_11_H_24_	156.31	3.5	98
Linoleic acid	28.21	C_18_H_32_O_2_	280.40	3.6	94
Stearic acid	28.47	C_18_H_36_O_2_	284.50	1.2	99
Trimethylsilane	19.47	C_3_H_10_Si	74.20	2.3	85
N-(trimethylsilyl) acetamide	8.35	C_5_H_13_NOSi	131.25	2.7	98

Abbreviations: Conc. (%), percentage concentration; MF, molecular formula; MW, molecular weight; QP, quality peak; RT, retention time.

^a^EML = Extract of *T. procumbens*, *M. scaber*, *M. pruriens*, and *C. papaya* mixture leaves.

**Table 3:** Components and proximate composition analysis (g kg^−1^ diet on dry matterbasis) of diets containing different levels of EML.

Ingredients	EML^a^ levels (g kg^−1^)				
	0.0	2.0	4.0	6.0	8.0
Fishmeal (crude protein 65%)	200	200	200	200	200
Soybean meal (crude protein 44%)	300	300	300	300	300
Groundnut cake (crude protein 45%)	300	300	300	300	300
Maize (crude protein 9%)	110	110	110	110	110
Soybean oil	30	30	30	30	30
Starch	40	38	36	34	32
Premix^b^	20	20	20	20	20
EML	0	2	4	6	8
Total	1000	1000	1000	1000	1000
Proximate composition (g kg^−1^)					
Dry matter	932	932	932	932	932
Crude protein	406	405	408	406	405
Ether extract	104	101	103	102	103
Total ash	77	76	78	78	78
Crude fiber	113	109	110	110	110

^a^EML = Extract of *T. procumbens*, *M. scaber*, *M. pruriens*, and *C. papaya* mixture leaves.

^b^Premixes = HI-MIXAQUA (fish) each 1 kg contains as follows. Vitamin A, 4,000,000 International Unit (IU); vitamin D3: 8,00,000 IU; vitamin E, 40,000 IU; vitamin K3: 1600 mg; vitamin B1: 4000 mg; vitamin B2: 3000 mg; vitamin B6: 3800 mg; vitamin B12: 3 µg; nicotinic acid: 18,000 mg; pantothenic acid: 8000 mg; folic acid: 800 mg; biotin: 100 µg; choline chloride: 120,000 mg; iron: 8000 mg; copper: 800 mg; manganese: 6000 mg; zinc: 20,000 mg; iodine: 400 mg; selenium: 40 mg; vitamin C (coated): 60,000 mg; inositol: 10,000 mg; cobalt: 150 mg; lysine: 10,000 mg; methionine: 10,000 mg; antioxidant: 25,000 mg.

**Table 4:** Growth performance of heteroclarias fed dietary EML for 56 days.^1^

Parameter	EML^2^ levels (g/kg)					*p*-Value
	0	2	4	6	8	
IBW (g)	14.47 ± 0.05^a^	14.45 ± 0.06^a^	14.45 ± 0.06^a^	14.60 ± 0.27^a^	14.47 ± 0.05^a^	0.475
FBW (g)	95.52 ± 0.62^d^	133.90 ± 1.14^c^	159.85 ± 3.09^b^	166.23 ± 2.53^a^	157.85 ± 1.97^b^	<0.001
BWG (g)	81.05 ± 0.66^d^	119.45 ± 1.17^c^	145.40 ± 3.09^b^	151.63 ± 2.43^a^	143.38 ± 1.98^b^	<0.001
SGR (%/day)	3.37 ± 0.02^d^	3.98 ± 0.02^c^	4.29 ± 0.04^ab^	4.35 ± 0.03^a^	4.27 ± 0.02^b^	<0.001
FI (g)	132.26 ± 3.41^d^	181.34 ± 7.37^c^	212.48 ± 6.52^b^	224.01 ± 3.03^a^	217.01 ± 3.74^ab^	<0.001
FCR	1.63 ± 0.03^a^	1.52 ± 0.05^b^	1.46 ± 0.01^b^	1.45 ± 0.02^b^	1.51 ± 0.02^b^	<0.001
SR (%)	893.33 ± 5.44^a^	98.33 ± 3.34^a^	98.33 ± 3.34^a^	98.33 ± 3.34^a^	95.00 ± 6.38^a^	0.405

*Note:* Values are presented as mean ± standard deviation. Means in the same row with difference superscript are statistically significantly different (*p*  < 0.05). SR, fish survival rate.

Abbreviations: BWG, body weight gain; FBW, final body weight; FCR, feed conversion ratio; FI, feed intake; IBW, initial body weight; SGR, specific growth rate.

^1^Number of replicates is 4.

^2^EML = Extract of *T. procumbens*, *M. scaber*, *M. pruriens*, and *C. papaya* mixture leaves.

**Table 5:** Digesta pH, viscosity, and short-chain fatty acids of heteroclarias fed diets containing various levels of EML for 56 days.^1^

Parameter	EML^2^ levels (g/kg)					*p*-Value
	0	2	4	6	8	
pH	7.79 ± 0.01^a^	7.62 ± 0.02^b^	7.50 ± 0.01^c^	7.50 ± 0.02^c^	7.41 ± 0.01^d^	<0.001
Viscosity	3.21 ± 2.01^a^	3.89 ± 0.03^a^	3.37 ± 0.18^a^	3.21 ± 0.01^a^	3.21 ± 0.01^a^	0.790
Acetic	9.81 ± 0.02^d^	11.23 ± 0.03^c^	14.33 ± 0.26^b^	14.84 ± 0.19^a^	14.59 ± 0.07^ab^	<0.001
Propionic	1.21 ± 0.01^a^	1.20 ± 0.01^a^	1.14 ± 0.02^b^	1.21 ± 0.01^a^	1.18 ± 0.05^ab^	0.005
Butyric	0.52 ± 0.02^c^	0.55 ± 0.01^c^	0.76 ± 0.01^b^	0.83 ± 0.02^ab^	0.86 ± 0.06^a^	<0.001

*Note:* Values are presented as mean ± standard deviation. Means in the same row with difference superscript are statistically significantly different (*p*  < 0.05).

Abbreviations: BASs, basophils; EOSs, eosinophils; Hb, hemoglobin; HETs, heterocytes; LYMs, lymphocytes; MONs, monocytes; PCV, packed cell volume; PLTs, platelets; RBCs, red blood cells; WBCs, white blood cells.

^1^Number of replicates is 4.

^2^EML = Extract of *T. procumbens*, *M. scaber*, *M. pruriens*, and *C. papaya* mixture leaves.

**Table 6:** Hematological parameter of heteroclarias fed diets containing various levels of EML for 56 days^1^.

Parameter	EML^2^ levels (g/kg)					*p*-Value
	0	2	4	6	8	
PCV (%)	19.75 ± 0.96^b^	20.25 ± 1.26^b^	26.50 ± 2.08^a^	27.00 ± 1.41^a^	26.00 ± 1.63^a^	<0.001
Hb (g/dL)	3.77 ± 0.06^d^	4.42 ± 0.10^c^	4.73 ± 0.05^b^	5.34 ± 0.05^a^	5.31 ± 0.05^a^	<0.001
RBC (× 10^6^/µL)	0.89 ± 0.08^b^	1.07 ± 0.03^ab^	1.05 ± 0.01^ab^	1.11 ± 0.01^a^	1.14 ± 0.01^a^	<0.001
WBC (× 10^3^/µL)	10.38 ± 0.63^c^	11.25 ± 0.65^bc^	11.50 ± 0.41^b^	12.50 ± 0.51^ab^	13.60 ± 0.64^a^	<0.001
PLT (× 10^6^/µL)	86.75 ± 5.38^d^	97.75 ± 7.93^d^	135.25 ± 5.85^c^	161.75 ± 8.18^b^	216.25 ± 12.45^a^	<0.001
LYM (%)	54.75 ± 1.71^c^	55.50 ± 2.08^c^	63.00 ± 1.63^b^	67.75 ± 2.75^ab^	70.75 ± 3.30^a^	<0.001
HET (%)	39.25 ± 1.71^a^	39.75 ± 1.89^a^	31.75 ± 2.06^b^	25.00 ± 3.16^c^	20.75 ± 3.50^c^	<0.001
MON (%)	2.00 ± 0.00^a^	1.50 ± 0.58^b^	2.75 ± 1.26^ab^	4.00 ± 0.82^a^	3.75 ± 0.50^a^	0.001
EOS (%)	3.50 ± 1.29^a^	2.75 ± 0.82^a^	1.75 ± 1.50^a^	2.75 ± 0.96^a^	4.00 ± 0.82^a^	0.103
BAS (%)	0.50 ± 0.05^a^	0.50 ± 0.05^a^	0.75 ± 0.05^a^	0.50 ± 0.04^a^	0.75 ± 0.09^a^	0.792

*Note:* Values are presented as mean ± standard deviation. Means in the same row with difference superscript are statistically significantly different (*p*  < 0.05).

Abbreviations: BASs, basophils; EOSs, eosinophils; Hb, hemoglobin; HETs, heterocytes; LYMs, lymphocytes; MONs, monocytes; PCV, packed cell volume; PLTs, platelets; RBCs, red blood cells; WBCs, white blood cells.

^1^Number of replicates is 4.

^2^EML = Extract of *T. procumbens*, *M. scaber*, *M. pruriens*, and *C. papaya* mixture leaves.

**Table 7:** Serum biochemistry profiles of heteroclarias fed diets containing various levels of EML for 56 days^1^.

EML^2^ levels (g/kg)	Parameter			
	UREA (mg/dL)	CREAT (mg/dL)	GLU (mg/dL)	CHOL (mg/dL)
0	10.35 ± 0.06^a^	1.30 ± 0.08^a^	232.25 ± 2.63^a^	170.50 ± 1.73^a^
2	10.03 ± 0.05^b^	1.18 ± 0.13^a^	222.50 ± 3.11^a^	162.50 ± 1.91^b^
4	9.76 ± 0.13^bc^	0.96 ± 0.05^b^	204.00 ± 12.70^b^	131.00 ± 2.71^c^
6	9.68 ± 0.12^c^	0.93 ± 0.02^b^	189.75 ± 3.75^c^	123.25 ± 2.22^d^
8	9.35 ± 0.24^d^	0.90 ± 0.01^b^	180.75 ± 0.96^c^	115.50 ± 1.92^e^
*p*-Value	<0.001	<0.001	<0.001	<0.001

*Note:* Values are presented as mean ± standard deviation. Means in the column row with difference superscript are statistically significantly different (*p*  < 0.05).

Abbreviations: CHOL, cholesterol; CREAT, creatinine; GLU, glucose; UREA, urea.

^1^Number of replicates is 4.

^2^EML = Extract of *T. procumbens*, *M. scaber*, *M. pruriens*, and *C. papaya* mixture leaves.

**Table 8:** Antioxidants profiles of heteroclarias fed diets containing various levels of EML for 56 days^1^.

EML^2^ levels (g/kg)	Parameter		
	SOD (IU/mL)	CAT (IU/mL)	MDA (nmol/L)
0	0.75 ± 0.34^d^	2.79 ± 0.11^d^	5.20 ± 0.11^a^
2	1.62 ± 0.31^c^	3.05 ± 0.23^cd^	4.16 ± 0.13^b^
4	2.36 ± 0.31^b^	3.72 ± 0.26^bc^	3.42 ± 0.19^c^
6	3.46 ± 0.15^a^	4.16 ± 0.40^b^	2.72 ± 0.18^d^
8	3.81 ± 0.10^a^	5.56 ± 0.56^a^	2.33 ± 0.16^e^
*p*-Value	<0.001	<0.001	<0.001

*Note:* Values are presented as mean ± standard deviation. Means in the column row with difference superscript are statistically significantly different (*p*  < 0.05).

Abbreviations: CAT, catalase; MDA, malondialdehyde; SOD, superoxide dismutase.

^1^Number of replicates is 4.

^2^EML = Extract of *T. procumbens*, *M. scaber*, *M. pruriens*, and *C. papaya* mixture leaves.

## Data Availability

The data that support the findings of this study are available in the Supporting Information of this article.

## Supporting Information

Supporting Information Raw data for dietary EML manuscript.

## References

[1] FAO (2024). *The State of World Fisheries and Aquaculture 2024*.

[2] TA A., OH A. (2022). Growth Performance and Serum Composition of Heteroclarias Fed, *Moringa oleifera*, Leaf Meal-Based Diet. *International Journal of Fisheries and Aquatic Studies*.

[3] FAO (2021). FAO Yearbook. Fishery and Aquaculture Statistics 2019/FAO Annuaire. Statistiques des pêches et de l’aquaculture 2019/FAO anuario. *Estadísticas de pesca y acuicultura*.

[4] FAO (2022). FAO Yearbook. Fishery and Aquaculture Statistics 2020/FAO annuaire. Statistiques des pêches et de l’aquaculture 2020/FAO anuario. *Estadísticas de pesca y acuicultura*.

[5] Adeshina I., Paray B. A., Bhat E. A. (2024). Dietary *β*-Mannanase Affects the Growth, Antioxidant, and Immunes Responses of African Catfish, *Clarias gariepinus*, and Its Challenge Against, *Aeromonas hydrophila*, Infection. *Aquaculture Nutrition*.

[6] Prikrylová I., Radim B., Gelnar M. (2012). *Gyrodactylus malalai* sp. nov.(Monogenea, Gyrodactylidae) From Nile Tilapia, *Oreochromis niloticus* (L.) and Redbelly Tilapia, *Tilapia zillii* (Gervais) (Teleostei, Cichlidae) in the Lake Turkana, Kenya. *Acta Parasitology*.

[7] Garcia-Vavijo A., Hansen H., Shinn A. P. (2007). A Revised Description of, *Gyrodactylus Cichlidarum*, Paperna, 1968 (Gyrodactylidae) From the Nile Tilapia, *Oreochromis niloticus* (Cichlidae), and Synonymy With D. Niloticus, Cone. *Folia Parasitology*.

[8] Gado M. S. M., Mahfouz N. B., Moustafa E. M. M., Lolo E. E. E. (2017). Prevalence of Some Ectoparasitic Diseases in African Catfish (*Clarias gariepinus*) at Kafr El-Sheikh Governorate. *International Journal of Fisheries and Aquatic Studies*.

[9] Akoll P., Konecny R., Mwanja W. W., Schiemer F. (2012). Risk Assessment of Parasitic Helminths on Cultured Nile Tilapia (Oreochromis niloticus, L.). *Aquaculture*.

[10] Adeshina I., Tiamiyu L. O., Akpoilih B. U., Jenyo-Oni A., Ajani E. K. (2021). Dietary, *Mitracarpus scaber*, Leaves Extract Improved Growth, Antioxidants, Non-Specific Immunity, and Resistance of Nile Tilapia, *Oreochromis niloticus* to, *Gyrodactylus malalai*, Infestation. *Aquaculture*.

[11] Ministry of Agriculture and Waters, Extension New Bulletin (2001). FRRC (Fisheries Resources Research Centre), “The Most Common Diseases in Fish Farms and Some Marine Fishes in the Eastern Region, Saudi Arabia.

[12] Jahanbakhshi A., Pourmozaffar S., Mozanzadeh M. T., Adeshina I., Zehra S., Vega-Heredia S. (2023). Dietary Effect of Grape Seed Proanthocyanidin Extract on Growth Performance, Serum Biochemical Parameters, Skin Mucosal Immune Response, and Antioxidant Capacity in Goldfish. *Annals of Animal Science*.

[13] Eissa E. S. H., Monier M. N., Abd El-Aziz Y. M. (2025). The Efficacy of Dietary Commercial Probiotic (*Bacillus subtilis*) on Growth Performance, Hemato-Biochemical Response, and Histological Status of Red Tilapia (*Oreochromis* sp.). *Journal of Applied Aquaculture*.

[14] Adeshina I., Jenyo-Oni O., Emikpe B. O., Ajani E. K., Abdel-Tawwab M. (2019). Stimulatory Effect of Dietary Clove, *Eugenia caryophyllata*, Bud Extract on Growth Performance, Nutrient Utilization, Antioxidant Capacity, and Tolerance of African Catfish, *Clarias gariepinus* (B.), to, *Aeromonas hydrophila*, Infection. *Journal of the World Aquaculture Society*.

[15] Abdel-Tawwab M., Mousa M. A. A., Mohammed M. A. (2010). Use of Live Baker’s Yeast, *Saccharomyces cerevisiae*, in Practical Diet to Enhance the Growth Performance of Galilee Tilapia, *Sarotherodon galilaeus* (L.), and Its Resistance to Environmental Copper Toxicity. *Journal of the World Aquaculture Society*.

[16] Abdel-Tawwab M., Adeshina I., Jenyo-Oni A., Ajani E. K., Emikpe B. O. (2018). Growth, Physiological, Antioxidants, and Immune Response of African Catfish, *Clarias gariepinus* (B.), to Dietary Clove Basil, *Ocimum gratissimum*, Leaf Extract and Its Susceptibility to, *Listeria monocytogenes*, Infection. *Fish & Shellfish Immunology*.

[17] N′Guessan P. A., Ouattara L. H., Ambeu-Loko N. C. M. (2024). Phytochemical Study and Antioxidant Activity of the Aerial Part of Mitracarpus Scaber Zucc. (Rubiaceae): A Medicinal Plant From Northern Côte d’Ivoire. *International Journal of Chemical Studies*.

[18] Adama A. A., Sanogo R., etal R. (2022). Cyclohexane and Ethyl Acetate Extracts of *Mitracarpus scaber* Zucc. (Rubiaceae) Show Potent Antimicrobial Activity Against Gram-Positive Bacteria and Fungi. *World Journal of Pharmaceutical Research*.

[19] de Queiroz M. N., dos Santos Torres Z. E., Pohlit A. M., Ono E. A., Affonso E. G. (2022). Therapeutic Potential Of, *Piper aduncum*, Leaf Extract in the Control of Monogeneans in Tambaqui (*Colossoma macropomum*). *Aquaculture*.

[20] Zhang X. P., Li W. X., Ai T. S., Zou H., Wu S. G., Wang G. T. (2014). The Efficacy of Four Common Anthelmintic Drugs and Traditional Chinese Medicinal Plant Extracts to Control *Dactylogyrus vastator* (Monogenea). *Aquaculture*.

[21] de Andrade J. I. A., Jerônimo G. T., Brasil E. M. (2016). Efficacy of Seed Extract of, *Bixa orellana*, Against Monogenean Gill Parasites and Physiological Aspects of, *Colossoma macropomum*, After Bath Treatment. *Aquaculture*.

[22] Ekanem A. P., Obiekezie A., Kloas W., Knopf K. (2004). Effects of Crude Extracts of *Mucuna pruriens* (Fabaceae) and *Carica papaya* (Caricaceae) Against the Protozoan Fish Parasite *Ichthyophthirius multifiliis*. *Parasitology Research*.

[23] Ling F., Wang J.-G., Lu C., Wang G.-X., Lui Y.-H., Gong X.-N. (2012). Effects of Aqueous Extract of *Capsicum frutescens* (Solanaceae) Against the Fish Ectoparasite *Ichthyophthirius multifiliis*. *Parasitology Research*.

[24] Zhang J., Liu Y. J., Tian L. X. (2013). Effects of Dietary Astaxanthin on Growth, Antioxidant Capacity and Gene Expression in Pacific White Shrimp *Litopenaeus vannamei*. *Aquaculture Nutrition*.

[25] Explore Foods Foodomics (2025). Efficacy of *Mucuna pruriens* (L.) DC. in Treating Diabetes, Parkinson’s Disease, and Erectile Dysfunction. *Explore Foods Foodomics*.

[26] Govindan S., Krishnamoorthy G. (2024). Seed Extract Stimulating Immune System in, *Litopenaeus vannamei*, Against *Vibrio harveyi*. *Aquaculture*.

[27] Alhaiqi N. S., Afifi S. M., Mahyoub J. A., Abdel-Gaber R. A., Delic D., Dkhil M. A. (2024). Anthelmintic Activity of Carica papaya Leaf Extracts: Insights from In Vitro and In Silico Investigations. *Combinatorial Chemistry & High Throughput Screening*.

[28] Zirintunda G., Kateregga J., Nalule S., Biryomumaisho S., Omujal F., Okwee-Acai J. (2025). Extracts of Carica papaya L. and Capsicum annuum L. showed Comparable Efficacy to Piperazine Citrate and Levamisole Hydrochloride In Treatment of Poultry Helminths. *Beni-Suef Journal of Basic and Applied Sciences*.

[29] Adeshina I., Abdel-Tawwab M., Tijjani Z. A., Tiamiyu L. O., Jahanbakhshi A. (2021). Dietary, *Tridax procumbens*, Leaves Extract Stimulated Growth, Antioxidants, Immunity, and Resistance of Nile Tilapia, *Oreochromis niloticus*, to Monogenean Parasitic Infection. *Aquaculture*.

[30] Mohamed Ahadu Shareef T. H., Navabshan I., Divan Masood M. M., Yuvaraj T. E., Sherif A. (2024). Computational Evaluation of, *Tridax procumbens*, Phytoconstituents for Their Anti-Inflammatory Potential Via Molecular Docking Against IL-1, IL-6, and TNF-*A* Targets. *Journal of Pharmaceutical and Biomedical Sciences*.

[31] Wankhade S. S., Shivani S. (2024). Review on Wound-Healing Properties of *Tridax procumbens*: Phytochemical Profile and Pharmacological Activities. *Indo American Journal of Pharmaceutical Sciences*.

[32] Adeshina I., Emikpe B. O., Jenyo-Oni A., Ajani E. K., Abubakar M. I. (2019). Growth Performance, Gut Morphometry and Innate Immune Profiles of Common Carp, *Cyprinus carpio*, Juveniles Fed Diet Fortified With, *Mitracarpus Scaber*, Leaves Extract and Its Susceptibility to Pathogenic Bacteria, *Aeromonas hydrophila*. *Acta Biologica*.

[33] Mayuri P. N. (2012). Screening of, *Ailanthus excelsa*, Roxb. for Secondary Metabolites. *Journal of Current Pharmaceuticals Research*.

[34] Odebiyi O. O., Sofowora E. A. (1978). Phytochemical Screening of Nigerian Medicinal Plants II. *Lloydia-the Journal of Natural Products*.

[35] Marcano L., Hasenawa D. (1991). Analysis of Phytochemicals in Leaves and Seeds. *Agronomy Journal*.

[36] Harborne J. B. (1998). *Phytochemical Methods: A Guide to Modern Techniques of Plant Analysis*.

[37] Joshi B., Sah G. P., Basnet B. B. (2011). Phytochemical Extraction and Antimicrobial Properties of Different Medicinal Plants*: Ocimum sanctum* (Tulsi), *Eugenia caryophyllata* (Clove), *Achyranthes bidentata* (Datiwan) and *Azadirachta indica* (Neem). *Journal of Microbiology and Antimicrobials*.

[38] Hema R., Kumaravel S., Sivasubramanian C. (2010). GC-MS Study on the Potentials of *Syzygium aromaticum*. *Researcher*.

[39] Bayoub K. T., Baibai D., Mountassif A., Retmane A., Soukri A. (2010). Antibacterial Activities of the Crude Ethanol Extracts of Medicinal Plants Against *Listeria monocytogenes* and Some Other Pathogenic Strains. *African Journal of Biotechnology*.

[40] Boyd C. E., Tucker C. S. (2012). *Pond Aquaculture Water Quality Management*.

[41] AVMA (2020). *AVMA Guidelines for the Euthanasia of Animals*.

[42] Bancroft J. D., Gamble M. (2008). *Theory and Practice of Histological Techniques”*.

[43] Eyarefe O. D., Emikpe B. O., Arowolo A. O. (2008). Small Bowel Responses to Enteral Honey and Glutamine Administration Following Massive Small Bowel Resection in Rabbit. *African Journal of Medical Science*.

[44] da Cruz T. P., Wernick B., Kozu A. Y. K. (2024). Dietary *β*-Mannanase Supplementation Decreases Digesta Viscosity, Improves Growth and Modulates Gut Microbiota in Juvenile Nile Tilapia, *Orechromis niloticus*, Fed a Soybean Meal-Based Diet. *Aquaculture*.

[45] Vankampen E. J., Ziglstra W. G. (1961). Colorimetric Determination of Hemoglobin. *Clinical Chemistry Acta*.

[46] Brown B. A. (1980). *Hematology: Principles and Procedures”*.

[47] Reitman S., Frankel S. (1957). A Colorimetric Method for the Determination of Serum Glutamic Oxalacetic and Glutamic Pyruvic Transaminases. *American Journal of Clinical Pathology*.

[48] Tietze L. F., von Kiedrowski G., Berger B. (2024). Intramolecular Cycloadditions. Part 4. Stereo- and Regioselective Syntheses of Enantiomerically Pure (+)- and (−)-Hexahydrocannabinol by Intramolecular Cycloaddition. *Chemischer Informationsdienst*.

[49] Ajeniyi S. A., Solomon R. J. (2014). Urea and Creatinine of *Clarias gariepinus* in Three Different Commercial Ponds. *Nature and Science*.

[50] Osuigwe D. I., Obiekezie A. I., Onuoha G. C. (2005). Some Haematological Changes in Hybrid Catfish (*Heterobranchus longifilis* × *Clarias gariepinus*) Fed Different Dietary Levels of Raw and Boiled Jackbean (*Canavalia ensiformis*) Seed Meal. *African Journal of Biotechnology*.

[51] McCord J. M., Fridovich I. (1969). Superoxide Dismutase an Enzymic Function for Erythrocuprein (Hemocuprein). *Journal of Biological Chemistry*.

[52] Aebi H. (1984). Catalase in Vitro. *Methods in Enzymology*.

[53] Secombes C. J., Stolen J. S., Anderson D. P., Roberston B. S., van Muiswinkel W. B. (1990). Isolation of Salmonid Macrophages and Analysis of Their Killing Activity. *Techniques in Fish Immunology*.

[54] Grinde B. (1989). Lysozyme from Rainbow Trout, *Salmo gairdneri*, Richardson an Anti-Bacterial Agents Against Fish Pathogens. *Journal of Fish Diseases*.

[55] Stoskopf M. (2015). Biology and Management of Laboratory Fishes. *Laboratory Animal Medicine*.

[56] Dytham C. (2011). *Choosing and Using Statistics: A Biologist’s Guide*.

[57] Fawole F. J., Yisa R. O., Jayeoba O. O., Adeshina I., Ahmed A. O., Emikpe B. O. (2022). Effect of Dietary Polyherbal Mixture on Growth Performance, Haemato-Immunological Indices, Antioxidant Responses, and Intestinal Morphometry of African Catfish, *Clarias gariepinus*. *Aquaculture Nutrition*.

[58] Wang R. X., Yao T., Liu X. J., Wang J. Y. (2018). Isolation and Characterisation of *Vibrio harveyi* as Etiological Agent of Foot Pustule Disease in the Abalone, *Haliotis Discus*, Hannai Ino 1953. *Indian Journal of Fisheries*.

[59] Huang M., Lin H., Xu C. (2020). Growth, Metabolite, Antioxidative Capacity, Transcriptome, and the Metabolome Response to Dietary Choline Chloride in Pacific White Shrimp *Litopenaeus vannamei*. *Animals*.

[60] Raissy M., Ghafarifarsani H., Hoseinifar S. H., El-Haroun E. R., Naserabad S. S., Van Doan H. (2022). The Effect of Dietary Combined Herbs Extracts (Oak Acorn, Coriander, and Common Mallow) on Growth, Digestive Enzymes, Antioxidant and Immune Response, and Resistance Against, *Aeromonas Hydrophila*, Infection in Common Carp, *Cyprinus carpio*. *Aquaculture*.

[61] Ji S.-C., Jeong G.-S., Gwang-Soon I. M., Lee S.-W., Yoo J.-H., Takii K. (2007). Dietary Medicinal Herbs Improve Growth Performance, Fatty Acid Utilization, and Stress Recovery of Japanese Flounder. *Fisheries Science*.

[62] Balahbib A., Omari N. El, Hachlafi N. E. L. (2021). Health Beneficial and Pharmacological Properties of P-Cymene. *Food Chemistry and Toxicology*.

[63] Dimitroglou A., Merrifield D. L., Spring P., Sweetman J., Moate R., Davies S. J. (2010). Effects of Mannanoligosaccharide (MOS) Supplementation on Growth Performance, Feed Utilisation, Intestinal Histology, and Gut Microbiota of Gilthead Seabream (*Sparus Aurata*). *Aquaculture*.

[64] Zhou Q.-C., Buentello J. A., Gatlin D. M. (2010). Effects of Dietary Prebiotics on Growth Performance, Immune Response and Intestinal Morphology of Redrum (*Sciaenops Ocellatus*). *Aquaculture*.

[65] Zahran E., Risha E., AbdelHamid F., Mahgoub H. A., Ibrahim T. (2014). Effects of Dietary Astragalus Polysaccharides (APS) on Growth Performance, Immunological Parameters, Digestive Enzymes, and Intestinal Morphology of Nile Tilapia (*Oreochromis Niloticus*). *Fish and Shellfish Immunology*.

[66] Zhang H. Y., Xuan L., Xi W. Q. (2010). Effects of Astragalus Polysaccharide on Structure of Intestinal Villus and Intestinal Immunocyte of Tilapia. *Chinese Journal of Animal Nutrition*.

[67] Flint H. J., Duncan S. H., Scott K. P., Louis P. (2015). Links Between Diet, Gut Microbiota Composition and Gut Metabolism. *Proceedings of the Nutrition Society*.

[68] Ríos-Covián D., Ruas-Madiedo P., Margolles A. (2016). Intestinal Short Chain Fatty Acids and Their Link With Diet and Human Health. *Frontier Microbiology*.

[69] Robles R., Lozano A. B., Sevilla A., Márquez L., Nuez Ortín W., Moyano F. J. (2013). Effect of Partially Protected Butyrate Used as Feed Additive on Growth and Intestinal Metabolism in Sea Bream (*Sparus aurata*). *Fish Physiology and Biochemistry*.

[70] Ebrahimi M., Daeman N. H., Chong C. M. (2017). Comparing the Effects of Different Dietary Organic Acids on the Growth, Intestinal Short-Chain Fatty Acids, and Liver Histopathology of Red Hybrid Tilapia (Oreochromis sp.) and Potential Use of These as Preservatives. *Fish Physiology and Biochemistry*.

[71] Estensoro I., Ballester-Lozano G., Benedito-Palos L. (2016). Dietary Butyrate Helps to Restore the Intestinal Status of a Marine Teleost (*Sparus aurata*) Fed Extreme Diets Low in Fish Meal and Fish Oil. *PLoS ONE*.

[72] Tian L., Zhou X. Q., Jiang W. D. (2017). Sodium Butyrate Improved Intestinal Immune Function Associated With NF *κ*B and p38MAPK Signalling Pathways in Young Grass Carp (*Ctenopharyngodon idella*). *Fish & Shellfish Immunology*.

[73] Rimoldi S., Gliozheni E., Ascione C., Gini E., Terova G. (2018). Effect of a Specific Composition of Short- and Medium Chain Fatty Acid 1-Monoglycerides on Growth Performances and Gut Microbiota of Gilthead Sea Bream (*Sparus aurata*). *PeerJ*.

[74] Abarike E. D., Jian J., Tang J., Cai J., Sakyi E. M., Kuebutornye F. K. A. (2020). A Mixture of Chinese Herbs and A Commercial Probiotic *Bacillus* Species Improves Hemato-Immunological, Stress, and Antioxidant Parameters, and Expression Of HSP70 and HIF-1*α* Mrna To Hypoxia, Cold, and Heat Stress in Nile Tilapia, *Oreochromis niloticus*. *Aquaculture Reports*.

[75] Zhang Y., Li X., Wang Q. (2023). The Effects of Herbal Extracts on Serum Biochemistry and Hepatic Function in Nile Tilapia (*Oreochromis Niloticus*). *Aquaculture Reports*.

[76] Nguyen V. T., Pham H. D., Le T. N. (2023). Influence of Dietary Turmeric Extract on Metabolic Parameters and Immune Response in *Pangasius bocourti*. *Journal of Fish Biology*.

[77] Ogbe R. J., Affiku J. P., Amuta E. U. (2023). Phytochemical Composition and Antioxidant Properties of, *Tridax procumbens*, Extracts: Implications For Oxidative Stress Management. *Journal of Medicinal Plants Research*.

[78] Yuan Z., Zhu H., Li X., etal X. (2023). Antioxidant Effects of, *Carica papaya*, Leaf Extract Against Oxidative Stress-Induced Cellular Damage. *Plants*.

[79] Kumar R., Yadav A., Meena A. (2024). Therapeutic Potential of, *Mucuna pruriens*, In Managing Oxidative Stress-Related Disorders. *Antioxidants*.

[80] Olorunnisola O. S., Ojo O. O. (2023). Bioactive Compounds of Mitracarpus scaber With antioxidant properties: A Review. *Journal of Herbal Medicine*.

[81] Abdel-Latif H. M. R., Abdel-Tawwab M., Khafaga A. F., Dawood M. A. O. (2020). Dietary Origanum Essential Oil Improved Antioxidative Status, Immune-Related Genes, and Resistance of Common Carp (*Cyprinus carpio* L.) to, *Aeromonas hydrophila*, Infection. *Fish and Shellfish Immunology*.

[82] Fu Y., Zhang Q., Xu D. H. (2014). Parasiticidal Effects of Morus Alba Root Bark Extracts Against, *Ichthyophthirius multifiliis*, Infecting Grass Carp. *Diseases of Aquatic Organism*.

[83] Liu Y.-M., Zhang Q.-Z., Xu D.-H. (2017). Antiparasitic Efficacy of Curcumin From *Curcuma longa* Against *Ichthyophthirius multifiliis* in Grass Carp. *Veterinary Parasitology*.

